# Crystal Structures of Malonyl-Coenzyme A Decarboxylase Provide Insights into Its Catalytic Mechanism and Disease-Causing Mutations

**DOI:** 10.1016/j.str.2013.05.001

**Published:** 2013-07-02

**Authors:** D. Sean Froese, Farhad Forouhar, Timothy H. Tran, Melanie Vollmar, Yi Seul Kim, Scott Lew, Helen Neely, Jayaraman Seetharaman, Yang Shen, Rong Xiao, Thomas B. Acton, John K. Everett, Giuseppe Cannone, Sriharsha Puranik, Pavel Savitsky, Tobias Krojer, Ewa S. Pilka, Wasim Kiyani, Wen Hwa Lee, Brian D. Marsden, Frank von Delft, Charles K. Allerston, Laura Spagnolo, Opher Gileadi, Gaetano T. Montelione, Udo Oppermann, Wyatt W. Yue, Liang Tong

**Affiliations:** 1 Structural Genomics Consortium, University of Oxford, Oxford OX3 7DQ, UK; 2 Department of Biological Sciences, Northeast Structural Genomics Consortium, Columbia University, New York, NY 10027, USA; 3 Center for Advanced Biotechnology and Medicine, Department of Molecular Biology and Biochemistry, Rutgers University, Piscataway, NJ 08854, USA; 4 Department of Biochemistry, Northeast Structural Genomics Consortium, Robert Wood Johnson Medical School, Piscataway, NJ 08854, USA; 5 Institute of Structural Molecular Biology, University of Edinburgh, Edinburgh EH9 3JR, UK; 6 NIHR Oxford Biomedical Research Unit, Botnar Research Centre, Oxford OX3 7LD, UK

## Abstract

Malonyl-coenzyme A decarboxylase (MCD) is found from bacteria to humans, has important roles in regulating fatty acid metabolism and food intake, and is an attractive target for drug discovery. We report here four crystal structures of MCD from human, *Rhodopseudomonas palustris*, *Agrobacterium vitis*, and *Cupriavidus metallidurans* at up to 2.3 Å resolution. The MCD monomer contains an N-terminal helical domain involved in oligomerization and a C-terminal catalytic domain. The four structures exhibit substantial differences in the organization of the helical domains and, consequently, the oligomeric states and intersubunit interfaces. Unexpectedly, the MCD catalytic domain is structurally homologous to those of the GCN5-related *N*-acetyltransferase superfamily, especially the curacin A polyketide synthase catalytic module, with a conserved His-Ser/Thr dyad important for catalysis. Our structures, along with mutagenesis and kinetic studies, provide a molecular basis for understanding pathogenic mutations and catalysis, as well as a template for structure-based drug design.

## Highlights

• Structures of human and bacterial MCDs were determined at up to 2.3 Å resolution
• Distinct tetrameric and dimeric MCD oligomerizations were observed
• Unexpected homology to the GNAT superfamily gives insights into catalytic mechanism
• The structures provide the molecular basis for the disease-causing mutations in MCD

## Teaser

Malonyl-CoA decarboxylase (MCD) is important in fatty acid metabolism. Froese et al. report structures of several MCDs and show that the MCD catalytic domain shares structural homology with GNAT superfamily. The structures further our understanding of catalysis, pathogenic mutations, and drug design.

## Introduction

Malonyl-coenzyme A (malonyl-CoA) has long been established as the key intermediate in the biosynthesis of long-chain and very long-chain fatty acids (Wakil et al., 1983; Zammit, 1999), and it also has a crucial role in the regulation of fatty acid oxidation in mammals through its potent inhibition of carnitine palmitoyltransferase I (McGarry and Brown, 1997; Ramsay et al., 2001). Recent studies have demonstrated other important functions for this metabolite (Folmes and Lopaschuk, 2007; Lopaschuk et al., 2010; Saggerson, 2008), for example, in the regulation of food intake through its actions in the central nervous system (Fantino, 2011; Lane et al., 2008; Wolfgang and Lane, 2008) and in the control of fuel selection (carbohydrate versus fatty acids) in many tissues (Folmes and Lopaschuk, 2007; Saggerson, 2008). Therefore, malonyl-CoA may be a crucial regulator of energy homeostasis.

Cellular malonyl-CoA levels are controlled by several enzymes. Malonyl-CoA is produced by acetyl-CoA carboxylase (Cronan and Waldrop, 2002; Tong, 2013; Wakil et al., 1983) and is consumed by fatty acid synthase (Kuhajda, 2006), elongases (Guillou et al., 2010), and malonyl-CoA decarboxylase (MCD, E.C. 4.1.1.9) (Saggerson, 2008). The functional importance of malonyl-CoA suggests that modulators of these enzymes may have therapeutic applications. Hepatic overexpression of MCD in rats led to a decrease in circulating free fatty acid and, more importantly, alleviated insulin resistance normally induced by a high-fat diet (An et al., 2004). On the other hand, inhibition of MCD in the heart may be beneficial for treating cardiac ischemia and reperfusion (Ussher and Lopaschuk, 2009), which is supported by observations on MCD^−/−^ mice (Dyck et al., 2006), as well as a collection of MCD inhibitors (Cheng et al., 2006a, 2006b, 2006c; Wallace et al., 2007). MCD inhibition has been found to be toxic to cancer cells, suggesting that it may be a target for anticancer therapy (Zhou et al., 2009). MCD inhibition can also reduce food intake and may be beneficial for obesity and diabetes treatment (Lopaschuk et al., 2010; Tang et al., 2010).

In mammals, MCD activity is found in the cytoplasm, mitochondria, and peroxisomes, and these different isoforms are encoded by a single gene (Courchesne-Smith et al., 1992; Gao et al., 1999; Joly et al., 2005; Sacksteder et al., 1999). MCD deficiency in humans (Mendelian Inheritance in Man No. 248360), a rare autosomal recessive disorder, is characterized by malonic aciduria, developmental delay, cardiomyopathy, and neonatal death in severe cases (Malvagia et al., 2007; Salomons et al., 2007; Xue et al., 2012), supporting the important role of this enzyme in cellular functions. There is, as yet, no genotype-phenotype correlation for the ∼30 pathogenic mutations identified (Xue et al., 2012).

**Figure 1:**
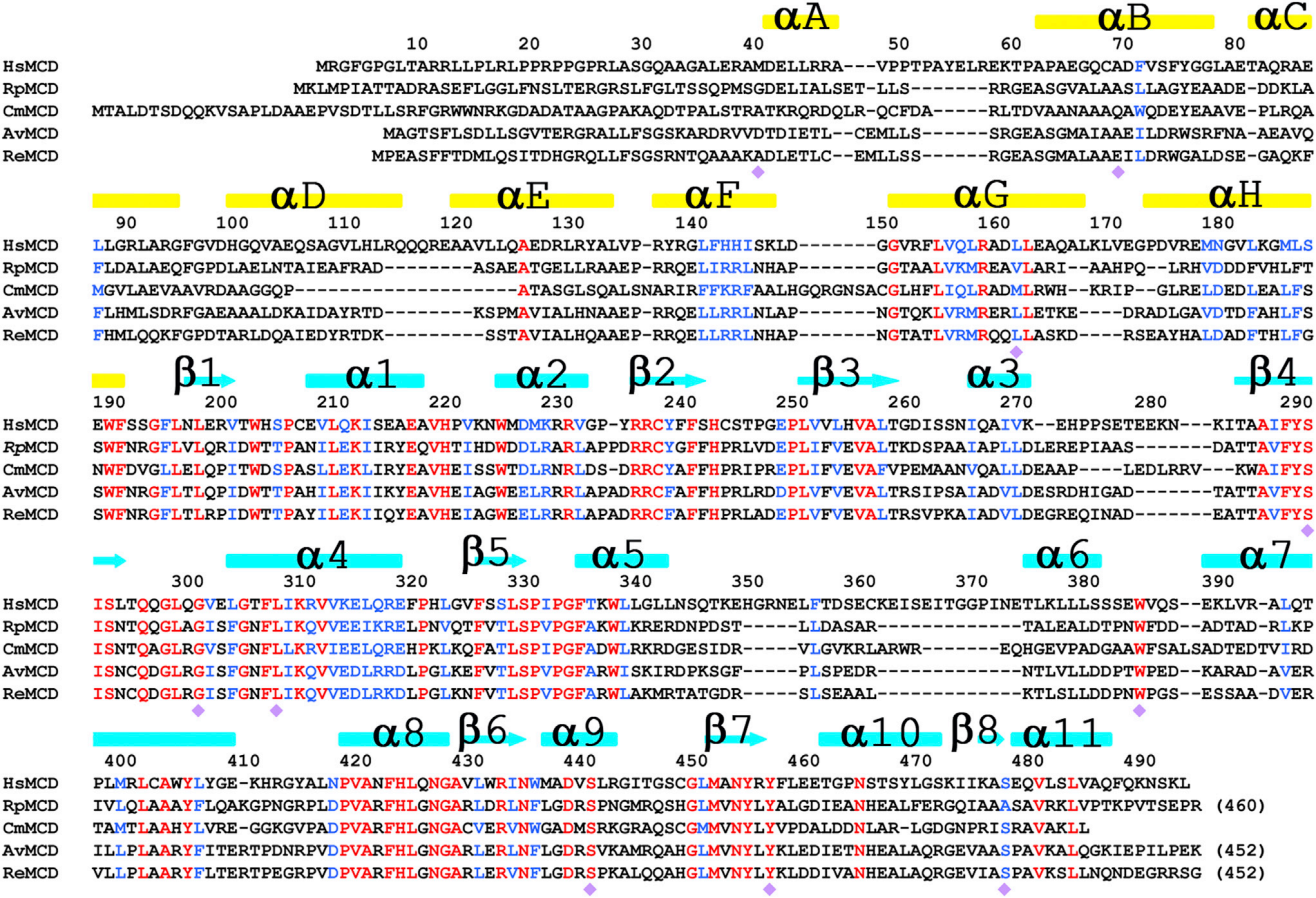
Sequence Alignment of HsMCD, RpMCD, CmMCD, AvMCD, and ReMCD The secondary structure elements for HsMCD are indicated at the top of the alignment, colored in yellow for those in the helical domain and cyan for those in the catalytic domain. Strictly conserved residues among the five sequences are shown in red and highly conserved residues in blue. The purple diamonds indicate sites of disease-causing missense mutations in HsMCD.

MCD (∼50 kDa) is also found in bacteria, plants, and other organisms with conserved amino acid sequences (Figure 1). For example, human MCD (HsMCD) and *Rhodopseudomonas palustris* MCD (RpMCD) share 34% sequence identity, while RpMCD and *Rhizobium etli* MCD (ReMCD) share 56% sequence identity (Figure 1). MCDs belong to the PFAM domain family PF05292 but do not share recognizable homology with other proteins in the sequence database, including methylmalonyl-CoA decarboxylase (Benning et al., 2000) and other decarboxylases. Purification of several animal and bacterial MCDs have been reported over the years (Kim and Kolattukudy, 1978; Kolattukudy et al., 1981; Lee et al., 2002; Lo et al., 2008; Zhou et al., 2004), and the crystallization of a bacterial MCD was also reported (Jung et al., 2003). However, no crystal structure was available on any of the MCDs, and the catalytic mechanism is still poorly understood.

We report here the crystal structures of human MCD as well as three bacterial MCDs at up to 2.3 Å resolution. The MCD monomer contains an N-terminal helical domain and a C-terminal catalytic domain, and the catalytic domain shares unexpected structural homology to the GCN5-related *N*-acetyltransferase (GNAT) superfamily. The N-terminal helical domain is involved in the oligomerization of MCDs, although there are substantial differences in the organization of the dimers and tetramers among MCD orthologs.

## Results and Discussion

### Structure Determination

Wild-type HsMCD (residues 40–491, corresponding to the mature mitochondrial form) failed to crystallize. Adopting the surface entropy reduction (SER) strategy (Cooper et al., 2007), two charged patches in HsMCD, Glu58-Lys59 and Glu278-Glu279-Lys280, were predicted to be surface-exposed by the SER prediction server (http://services.mbi.ucla.edu/SER/; Goldschmidt et al., 2007), and site-directed mutagenesis was used to substitute alanine for each of these residues simultaneously. The structure of the E58A/K59A/E278A/E279A/K280A quintuple mutant was determined by single isomorphous replacement with anomalous scattering and refined at 2.8 Å resolution (Table 1; Figure S1 available online). The mutant exhibited similar oligomeric and enzymatic properties as wild-type HsMCD (Table 2). Inspection of the structure revealed both alanine-substituted patches to be located in surface-exposed regions: Glu58-Lys59 was found in the loop connecting helices αA and αB, while the loop containing residues 278–280, connecting strands β3 and β4, was disordered.

Bacterial MCDs were targeted as part of the broad program of the National Institutes of Health (NIH) Protein Structure Initiative on structural coverage of large protein domain families (Liu et al., 2007). We obtained crystals for several bacterial MCDs, but most of them showed poor diffraction quality (about 5 Å resolution). After significant efforts at optimization and diffraction screening, we collected X-ray diffraction data for RpMCD, *Agrobacterium vitis* MCD (AvMCD), and *Cupriavidus metallidurans* MCD (CmMCD) at up to 2.3 Å resolution. We solved the structure of RpMCD by the selenomethionyl single-wavelength anomalous diffraction method and the structures of AvMCD and CmMCD by molecular replacement (Table 1).

### Structures of MCD Monomers

**Figure 2:**
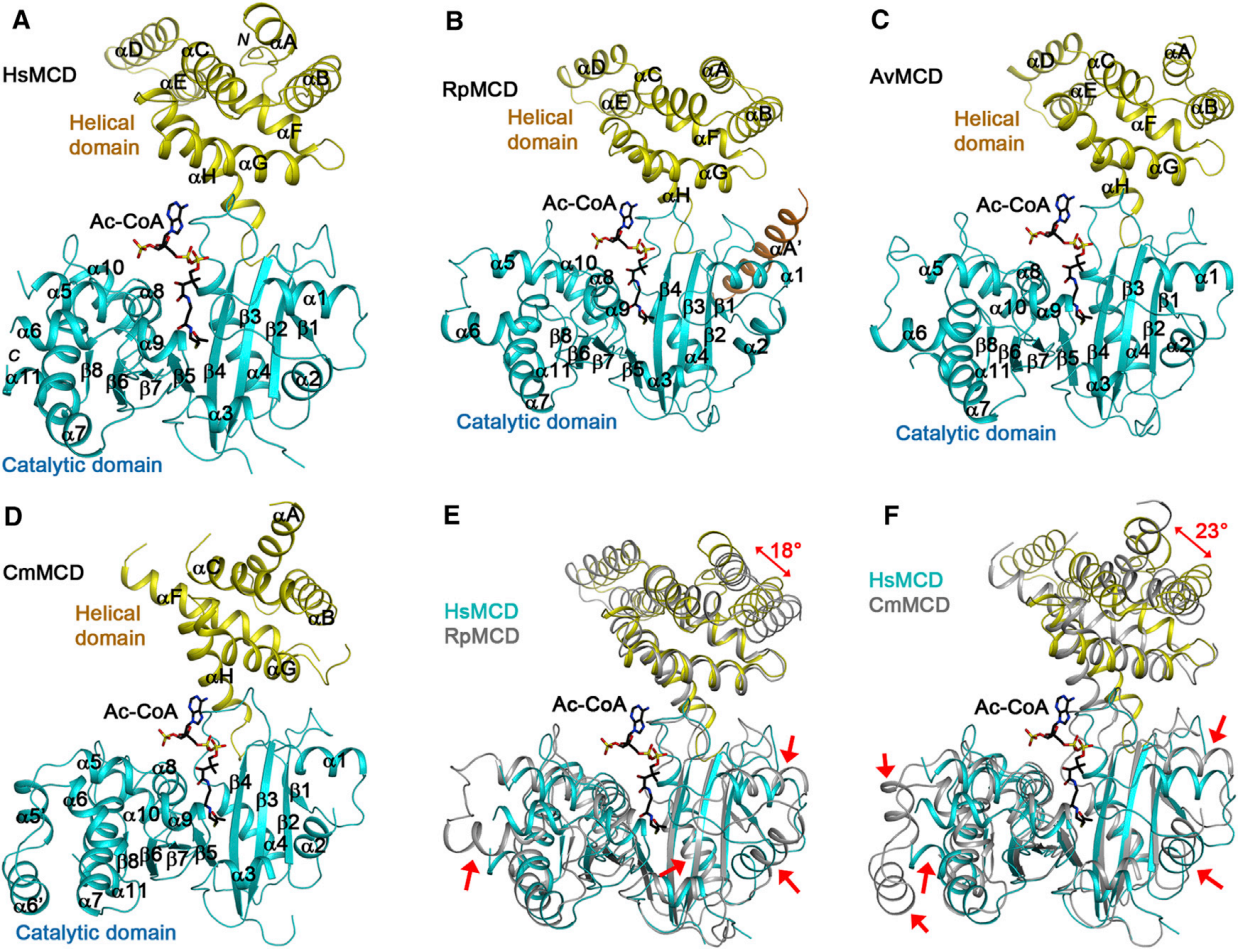
Crystal Structures of MCD Monomer Schematic drawing of the structures of HsMCD (A), RpMCD (B), AvMCD (C), and CmMCD (D). The N-terminal helical domain is shown in yellow and the C-terminal catalytic domain in cyan. The bound position of acetyl-CoA in CurA (Gu et al., 2007) is shown as a stick model (in black). Overlays of the structures of HsMCD (in color) and RpMCD (in gray) (E) and HsMCD (in color) and CmMCD (in gray) (F). Regions of structural difference in the catalytic domain are highlighted with the red arrows. The difference in the orientations of the helical domains is also indicated. The structure figures were produced with PyMOL (http://www.pymol.org). See also Figure S1.

The structures of the monomers of HsMCD (Figure 2A), RpMCD (Figure 2B), AvMCD (Figure 2C), and CmMCD (Figure 2D) can be divided into two domains: an N-terminal helical domain (130–150 residues) and a C-terminal catalytic domain (270–300 residues) connected via a short linker peptide. Consistent with this two-domain organization, the sequence conservation among the MCDs also appears to be bipartite (Figure 1). For example, the catalytic domains of HsMCD and RpMCD share 40% sequence identity, while their helical domains have only 24% identity. The N-terminal domain of HsMCD and several other MCDs are rich in Leu residues, which are concentrated in the helical segments.

The helical domain contains a bundle of six helices (αA–αC, αF–αH; Figures 2A–2D and S2). Helices αA and αB, and αG and αH form antiparallel hairpins and are arranged somewhat similar to those in armadillo/Huntington, elongation factor 3, protein phosphatase 2A, the yeast kinase TOR1 (HEAT), and tetratricopeptide repeats. However, the intervening helices αC and αF are located away from each other and run almost perpendicular to the other four helices. In addition, there is an insert of a helical hairpin (αD and αE) between helices αC and αF, which projects ∼30 Å away from the rest of the monomer (Figure S1). This helical hairpin insert as well as the helical domain itself helps mediate the oligomerization of MCD (see below).

The catalytic domain of MCD contains a central eight-stranded, mostly antiparallel β sheet (β1–β8) that is surrounded by at least 11 α helices (α1–α11; Figures 2A–2D). Strands β4 and β5 in the middle of the β sheet, the only two neighboring strands that are parallel to each other (in a β-α-β motif), are splayed apart from each other at their C-terminal ends, and the active site of the enzyme is located in this region (see below). There is an insert of three additional helices (α5–α7) between strands β5 and β6 in HsMCD, RpMCD, and AvMCD, while CmMCD has an insert of five helices here. The sequences of this insert are poorly conserved among the MCDs (Figure 1).

The overall structures of the catalytic domains are similar, with root-mean-square (rms) distance of 1.2–1.5 Å for equivalent Cα atoms located within 3 Å of each other between any pair of the four structures. This structural similarity is particularly high for the central β sheet of the catalytic domain, as illustrated for overlays between HsMCD and RpMCD (Figure 2E), HsMCD and CmMCD (Figure 2F), and other structure pairs (Figure S1). On the other hand, many of the helices of the catalytic domain, especially those in the insert between β5 and β6, have large positional differences. Moreover, with the catalytic domains in overlay, significant differences in the orientation and position of the N-terminal helical domain are observed among the MCDs, corresponding to relative rotations of 15°–25° (Figures 2E, 2F, and S3). In addition, the helical hairpin insert between αC and αF is absent in CmMCD (Figures 2D and S2).

### Oligomeric Architectures of MCDs

**Figure 3:**
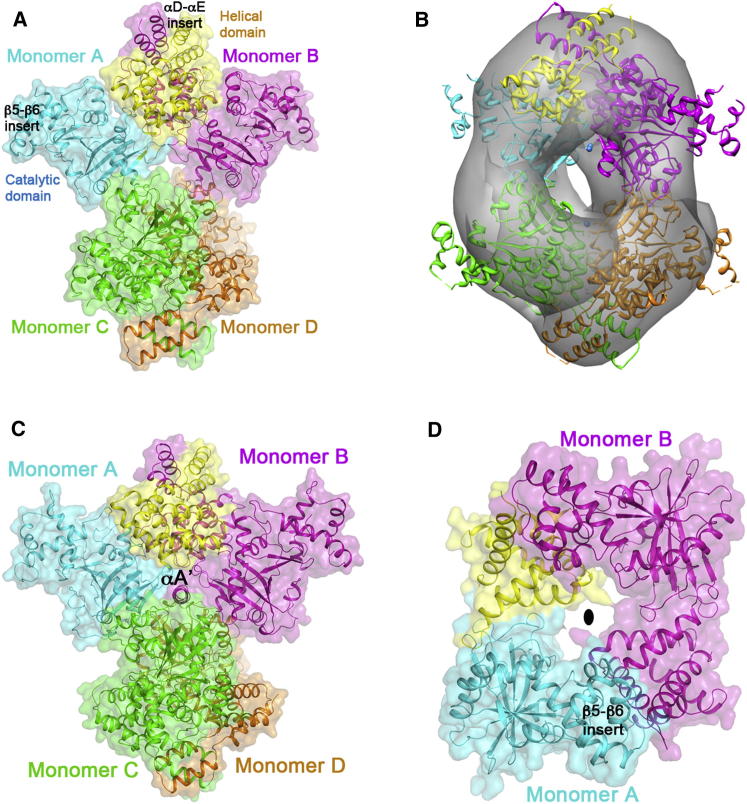
The Oligomers of MCD (A) Structure of the HsMCD tetramer. A semitransparent surface of the structure is also shown. (B) Docking of the HsMCD tetramer structure into the EM reconstruction. (C) Structure of the RpMCD tetramer. (D) Structure of the CmMCD dimer. The 2-fold axis of the dimer is indicated with the oval (black). See also Figure S2.

HsMCD is a tetramer in solution based on gel filtration chromatography and analytical ultracentrifugation (AUC) studies (Figure S2), consistent with the reported oligomerization state of many purified MCD enzymes. HsMCD sedimented in a single peak with an apparent molecular weight of ∼200 kDa (Figure S2). The HsMCD crystal structure shows that the tetramer is made of a dimer of dimers (Figure 3A). A tight dimer of HsMCD is formed by extensive contacts of the helical domains of the two monomers, and the αD and αE helical inserts of the two monomers interact with each other in this dimer interface. Especially, helix αE of this insert contributes four leucine residues (122, 123, 129, and 133) to the interface. Approximately 1,800 Å^2^ of the surface area of each monomer is buried in the dimer. Two HsMCD dimers then associate with each other through their catalytic domains, at ∼60° angle for the planes of the two dimers (Figure S2), to form the tetramer with 222 symmetry. This interface primarily involves residues at the N-terminal end of the catalytic domain, burying ∼500 Å^2^ of the monomer surface area.

The architecture and shape of the HsMCD tetramer were also analyzed by electron microscopy coupled to single particle analysis. Images of negatively stained HsMCD contained a homogenous population of monodispersed single particles (Figure S2). Our three-dimensional (3D) reconstruction revealed a particle of 125 × 100 × 100 Å^3^ in size with a central cavity, consistent in dimension and shape with the crystallographic tetramer (Figure 3B).

RpMCD and AvMCD are also tetramers in solution, based on multiangle static light scattering studies (data not shown). Like HsMCD, the RpMCD (Figure 3C) and AvMCD (Figure S2) tetramers are also dimer of dimers. However, the relative orientations of the dimers are substantially different (Figure S2). The central cavity of RpMCD tetramer also contains a helical segment (αA′) from the N terminus of two of the monomers (Figure 3C; Supplemental Information).

Surprisingly, CmMCD is a dimer in solution and the crystal structure reveals a completely different mode of dimerization as compared to HsMCD, RpMCD, and AvMCD. The two CmMCD monomers associate in a head-to-tail fashion such that the N-terminal helical domain of one monomer is in contact with the C-terminal catalytic domain of the other monomer, including the helical insert between strands β5 and β6 (Figure 3D). Approximately 1,100 Å^2^ of the surface area of each monomer is buried in this dimer.

The variations in the oligomers of MCDs are likely due to the differences in the conformations of the N-terminal helical domains and the positions of these domains relative to the catalytic domains. For example, clear differences are visible between the HsMCD and RpMCD dimers (Figure S2), thereby affecting their tetramer formation. CmMCD lacks the helical insert in the helical domain and has two additional helices between β5 and β6 in the catalytic domain (Figure 2D), which may explain why it cannot form a similar dimer and tetramer as HsMCD or RpMCD.

While this paper was under review, a structure of HsMCD at 3.29 Å resolution was reported (Aparicio et al., 2013). The overall structures of the HsMCD monomers in the two reports are similar, with rms distance of 1.5 Å for 380 equivalent Cα atoms (Figure S2). There are recognizable differences in the organization of the dimer and tetramer between the two structures, although the overall architectures of the two tetramers are similar (Figure S2).

### Unexpected Structural Homology to GNAT Enzymes

The structure of the MCD catalytic domain unexpectedly shows strong homology to proteins belonging to the GNAT superfamily (Dyda et al., 2000; Neuwald and Landsman, 1997; Vetting et al., 2005), based on a Protein Data Bank (PDB) search with the program DaliLite (Holm et al., 2008). As the name indicates, most of these enzymes are *N*-acetyltransferases, a catalytic activity highly distinct from that of MCD. On the other hand, the overall backbone folds of these enzymes are homologous. GNAT proteins typically contain a seven-stranded β sheet, which corresponds to the first seven strands in the catalytic domain of MCD, with the splaying of the β4 and β5 strands a common feature among these structures. The sequence conservation between MCDs and these other GNAT members is, however, much lower, around 10% for structurally equivalent residues. As expected, the catalytic machinery in the active site is also distinct between MCD and the *N*-acetyltransferases.

**Figure 4:**
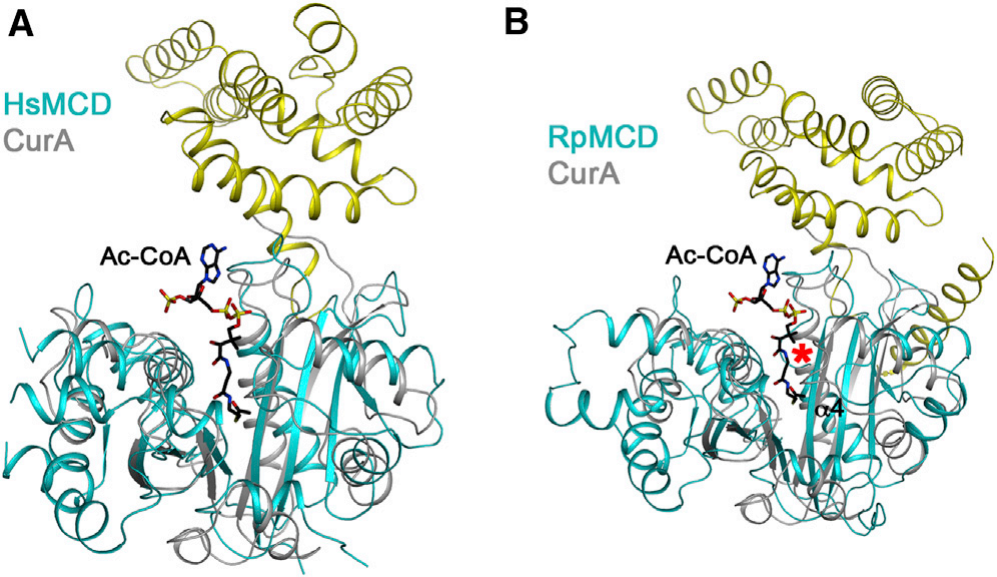
Structural Conservation with CurA (A) Overlay of the structures of HsMCD (in color) and CurA (in gray). Acetyl-CoA in the CurA complex is shown as a stick model (black). (B) Overlay of the structures of RpMCD (in color) and CurA (in gray). The red asterisk indicates large conformational differences in the N-terminal region of helix α4 between the two structures, which interacts with the phosphate groups of CoA in CurA.

The closest structural homolog, with a *Z* score of 16.6 from DaliLite, is the catalytic domain of the loading module of the polyketide synthase for curacin A (CurA) from *Lyngbya majuscula*, a GNAT protein that was shown not to have *N*-acetyltransferase activity (Gu et al., 2007; Figures 4A and 4B). Instead, this loading module harbors both malonyl-CoA decarboxylase and acetyl *S*-transferase activities. Despite the 13% identity for structurally equivalent residues between the two proteins, the catalytic residues for the decarboxylase activity of CurA are conserved in MCD (see below).

The N-terminal helical domain of MCDs does not have a counterpart in the GNAT enzymes. Consequently, the modes of oligomerization of MCDs are entirely different from these other GNAT enzymes. GNATs typically exist as monomers or dimerize via their GNAT core, and the predominant dimerization mode is by juxtaposing the GNAT β strands from both subunits to form a continuous β sheet. In contrast, the GNAT β strands in MCDs are not available for dimerization due to the presence of the large helical insert between strand β5 and β6. MCD dimerization is instead mediated by the N-terminal helical domain.

MCD represents a second example where a GNAT protein possesses a catalytic activity distinct from *N*-acetyltransferase. At the same time, the different activities of these GNAT proteins share the common substrate of acetyl- or malonyl-CoA. Therefore, the GNAT scaffold may have evolved to recognize the CoA moiety, and substitutions of several critical residues in the catalytic machinery may be sufficient to change the catalytic activity or substrate preference, such as succinyl-CoA (see below) (Vetting et al., 2008).

### The Active Site of MCD

Our extensive efforts to cocrystallize MCD with malonyl-CoA or acetyl-CoA have not been successful. Therefore, the structure of acetyl-CoA bound to CurA (Gu et al., 2007) was used as a guide for analyzing the MCD active site. This binding mode of acetyl-CoA is also generally similar to that in canonical GNAT enzymes, suggesting that the binding mode to MCD is likely to be similar as well.

**Figure 5:**
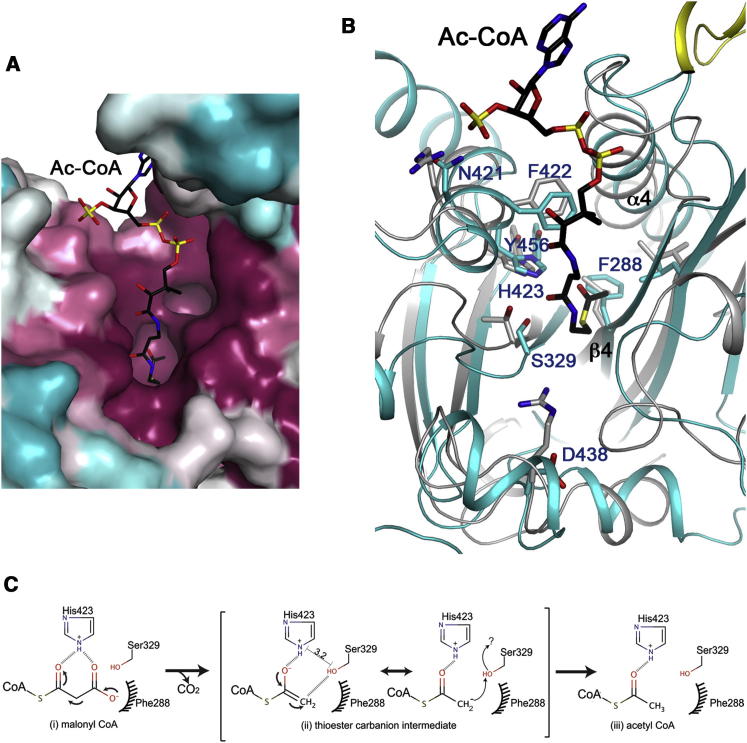
The Active Site of MCD (A) Molecular surface of HsMCD in the active site region, colored by sequence conservation (magenta, most conserved; cyan, least conserved). The bound position of acetyl-CoA in CurA (Gu et al., 2007) is shown as a stick model (in black). (B) An overlay of HsMCD (in color) and CurA (in gray) in the active site region. Side chains in HsMCD are labeled. The catalytic residues His423 and Ser329 of HsMCD are equivalent to His389 and Thr335 of CurA. Please see Figure S3 for a stereo version of this panel. (C) Proposed catalytic mechanism for MCD (HsMCD numbering). Interatomic distance between His423 imidazole nitrogen and Ser329 hydroxyl oxygen is denoted in black line. Question mark represents possible proton transfer to reprotonate Ser329, from His423, a water molecule, or other unidentified sources. See also Figure S3.

The active site of MCD is located in a prominent groove in the surface of the monomer, where the most conserved residues among these enzymes are located (Figure 5A). The other monomers of the MCD oligomer make little, if any, contribution to the active site. For RpMCD, residues 55–58 in the other monomer of the dimer, in the loop linking the first two helices of the N-terminal domain, approach within ∼10 Å of the expected position of the adenosine group in the active site. The equivalent loop in HsMCD is much longer, and Ala58 in this loop could have direct interactions with the adenine base of CoA. In the CmMCD dimer, the second monomer is located ∼20 Å away from the active site.

The pantotheine group of CoA is positioned along strand β4 (Figures 5B and S3). The diphosphate and adenosine groups interact with the loop linking this strand to the following helix (α4) in HsMCD, and the diphosphate group also has favorable interactions with the dipole of this helix. In fact, this loop contains the signature sequence motif A in canonical GNAT enzymes (Neuwald and Landsman, 1997), (Q/R)xxGx(G/A)xxL, but the motif is not fully conserved in MCD, 299-(Q/R/A)xxxx(G/A)xxL-307 (Figure 1). Moreover, the loop and the following helix α4 are positioned differently in RpMCD (Figures 4 and S3) and CmMCD (Figure S1), suggesting that the binding mode of CoA to these MCDs may be somewhat different unless there is a conformational change upon CoA binding in these two enzymes. The 3′ phosphate group on the ribose of CoA is recognized by Arg387 in CurA (equivalent to Asn421 in HsMCD; Figure 5B). This residue is equivalent to Arg387 in RpMCD, which may have a similar function. However, this Arg residue is not conserved among the MCD enzymes. It shows variations to Asn in animal MCDs and His in some bacterial MCDs (Figure 1).

The acetyl group of acetyl-CoA interacts with conserved residues His389 and Thr355 in CurA (Figure 5B), which is proposed to be the catalytic dyad for its malonyl-CoA decarboxylase activity (Gu et al., 2007). The H389A, H389N, and T355V mutants have drastically reduced decarboxylase activity. The equivalent residues, His423 and Ser329 in HsMCD and His389 and Ser312 in RpMCD, are strictly conserved among the MCDs (Figure 1). In comparison, the His residue is equivalent to a Tyr residue in the canonical GNAT enzymes, which serves as the general acid for catalysis (Dyda et al., 2000; Neuwald and Landsman, 1997; Vetting et al., 2005). On the other hand, the Thr/Ser residue of CurA/MCD is not conserved in the canonical GNAT enzymes, while the general base for these enzymes, a Glu residue, is not conserved in CurA/MCD. These differences in the catalytic residues are likely the molecular basis for the distinct activity of CurA/MCD compared to the canonical GNATs.

The imidazole ring of His389 in CurA is held in place through a hydrogen bond with Tyr419. The equivalent residue in HsMCD, Tyr456, is also conserved among the MCDs. The carboxylate group of the malonyl-CoA substrate may lie over the surface of Phe288 in strand β4 (Figure 5B; HsMCD numbering), which is another strictly conserved residue among the MCDs (Figure 1).

The main chain of Thr355 in CurA has interactions with Arg404. However, this Arg residue is not conserved in RpMCD (Asp404), and in fact, an Asp residue is conserved at this position among the MCDs. The Arg404 residue may also be important for the acetyl *S*-transferase activity of CurA (Gu et al., 2007). The absence of this residue in MCD may be consistent with its lack of *S*-transferase activity.

Acetylation of Lys210, as well as mutation of Lys210 to Met, has been reported to inactivate rat MCD (Nam et al., 2006). Binding of acetyl-CoA protects rat MCD from the acetylation. In the HsMCD structure, the equivalent Lys211 side chain is on the surface of the tetramer, in a helix (α1) connecting strands β1 and β2, and ∼20 Å from the active site. This side chain is mostly exposed to the solvent and does not have interactions with other conserved residues. Thus, it is not clear why this residue is essential for the catalysis by rat MCD.

To assess the functional importance of the active site His-Ser/Thr dyad of MCD, we carried out mutagenesis and kinetic studies with HsMCD. The S329A mutant of HsMCD had a 110-fold loss in *k*_cat_ and 58-fold loss in *k*_cat_/*K*_m_, and the H423N mutant had a 7-fold loss in *k*_cat_ (Table 2), consistent with their important roles in catalysis. In silico docking of malonyl-CoA into the HsMCD active site supports the kinetic data, showing that the substrate can position its thioester carbonyl (bridging the carboxylate leaving group and CoA backbone) in the vicinity (∼3.2 Å) of Ser329 and His423 (Figure S3).

The reaction mechanism for MCD bears similarity to the acetyl transfer reaction of canonical GNATs, as they all need to polarize and stabilize the developing negative charge on the thioester carbonyl group (Figure 5C). Using HsMCD as example, we postulate that MCD proceeds through the formation of the tautomerized enolate intermediate, with the Ser329 and His423 dyad adopting important catalytic roles consistent with our docking and kinetic analysis. Phe288 may provide a nonpolar environment for the CO_2_ leaving group, and the carbanion can abstract the proton from the side chain hydroxyl group of Ser329 acting as an acid (Figure 5C). This mechanism also has resemblance to that of a number of other CoA decarboxylases that do not employ cofactors (such as pyridoxal phosphate, thiamine, or metal ions) to delocalize the buildup of the negative charge (Fu et al., 2004).

### Molecular Basis of Disease-Causing Mutations in MCD

**Figure 6:**
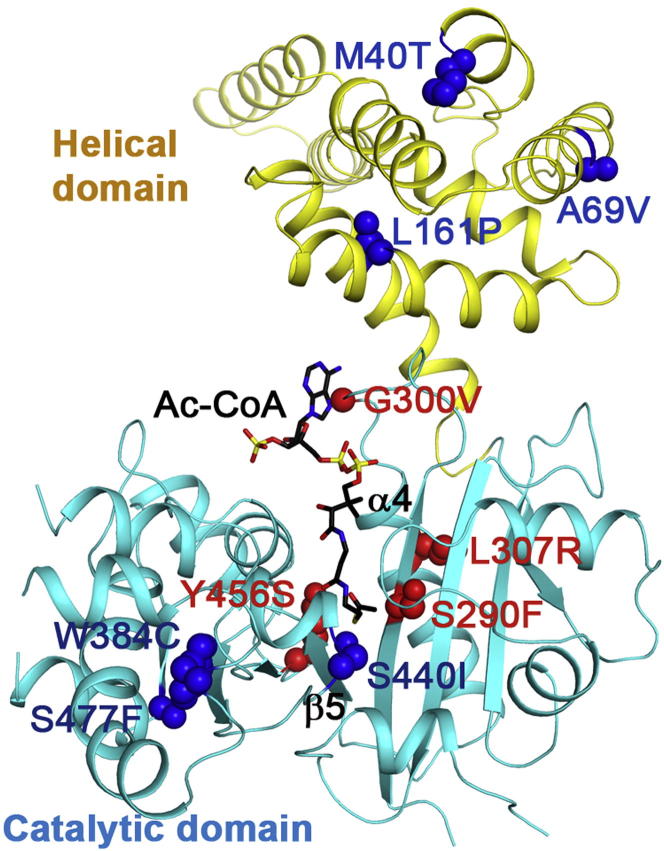
Molecular Basis for MCD Disease-Causing Mutations The 11 missense pathogenic mutations (in red for those that could affect catalysis/substrate binding and blue for those that could affect folding/stability) are mapped onto the structure of HsMCD. See also Table S1.

The structure of HsMCD provides a molecular framework for understanding the impact of loss-of-function alleles in hereditary MCD deficiency. While the nonsense, frameshift, and deletion mutations result in truncated and thus nonfunctional proteins, the 11 known missense mutations (Table S1) are distributed throughout the structure with no discernible hot spot regions (Figure 6). The potential structural and biochemical consequences of these substitutions can be classified into three types. The first type is protein mistargeting and includes the two most N-terminal mutations, G3D and M40T, each of which lies within the predicted mitochondrial targeting sequence. Both mutations have been demonstrated to affect protein localization (Wightman et al., 2003). The second type of substitution likely disrupts protein folding through either protein instability or aggregation. These include A69V and L161P in the N-terminal helical domain, as well as W384C, S440I, and S477F in the catalytic domain. The third type involves substitutions in the GNAT core, affecting residues highly conserved among MCDs. These include S290F, G300V, L307R, and Y456S (Figure 6). Ser290 is located in strand β4 near the binding site for the CoA pantotheine moiety, though facing away from it. Mutation to the larger Phe residue would be expected to result in clashes with neighboring amino acids (His254 and Tyr289) and, hence, possible rearrangement of the active site and a partial loss of function. Indeed, the reconstituted S290F mutant showed a 2-fold decrease in *k*_*cat*_ in vitro (Table 2). Gly300 and Leu307 are in the loop linking β4 and the following helix α4, being part of motif A. Both mutations result in substitution to larger residues that may clash with surrounding residues within this loop as well as residues on strand β3. Finally, Tyr456 interacts with the catalytic His423 residue (Figure 5C). Mutation to Ser would be expected to result in loss of His423 stabilization with consequent decreased substrate stability. In vitro, the Y456S mutant showed a 3.5-fold increased *K*_m_ (Table 2), consistent with this proposal.

In summary, we report here structural information on MCD, revealing its catalytic machinery, oligomer organization, mechanism of disease-causing mutations, as well as unexpected homology to GNAT enzymes. The structural information should also facilitate the design and optimization of inhibitors against this enzyme. It has been suggested that the current inhibitors may require a hydrogen bond to a histidine residue for binding (Cheng et al., 2006c), and our structure suggests that this very likely is the catalytic His423 residue. Therefore, the active site of MCD is a promising target for the development of new therapeutic agents against human diseases.

## Experimental Procedures

### Cloning, Expression, and Purification

A DNA fragment containing HsMCD (amino acids [aa] 40–491; IMAGE clone: 3357140) was subcloned into the pNIC28-Bsa4 vector (GenBank accession no. EF198106), incorporating an N-terminal tobacco etch virus (TEV)-cleavable His_6_-tag. For surface entropy reduction, residues Glu58-Lys59 and Glu278-Glu279-Lys280 were replaced with Ala. The expression plasmids were transformed into *E. coli* BL21(DE3)-R3-pRARE2 cells, grown in Terrific broth medium with induction by 0.1 mM isopropyl-β-D-thiogalactopyranoside (IPTG) overnight at 18°C. Protein was purified by affinity (Ni-nitrilotriacetic acid; QIAGEN) and gel filtration (Superdex 200; GE Healthcare) chromatography.

The production of the three bacterial MCDs, Rmet_2797 (CmMCD), RPA0560 (RpMCD), and Avi_5372 (AvMCD) from *Cupriavidus metallidurans*, *Rhodopseudomonas palustris*, and *Agrobacterium vitis*, respectively, was carried out as part of the high-throughput protein production process of the Northeast Structural Genomics Consortium (NESG) (Acton et al., 2005). The CmMCD, RpMCD, and AvMCD proteins correspond to NESG targets CrR76, RpR127, and RiR35, respectively. Full-length RpMCD and AvMCD were cloned into a pET21d (Novagen) derivative with C-terminal His-tag. Full-length CmMCD was cloned into pET26b with a C-terminal His-tag. *Escherichia coli* BL21 (DE3) pMGK cells, a rare codon enhanced strain, were transformed with each plasmid. A single isolate was transferred to 500 μl of Luria broth with ampicillin and kanamycin and incubated for 6 hr at 37°C. This preculture (40 μl) is then used to inoculate a 250 ml flask containing 40 ml of MJ9 minimal media (Jansson et al., 1996) and incubated overnight at 37°C. The entire volume of overnight culture is then used to inoculate a 2 l baffled flask containing 1.0 l of MJ9. The cultures are incubated at 37°C until the optical density at 600 nm reaches 0.8–1.0 units, equilibrated to 17°C, and induced with IPTG (1 mM final concentration) after addition of several amino acids to the medium to downregulate methionine synthesis (lysine, phenylalanine, and threonine at 100 mg/l; isoleucine, leucine, and valine at 50 mg/l; and L-selenomethionine at 60 mg/l) for 15 min (Doublié et al., 1996). In the case of CmMCD, the media contained methionine instead. Following overnight incubation, the cells were harvested by centrifugation. However, the full-length CmMCD, RpMCD, and AvMCD could not be purified this way, due to low expression and/or low solubility. Subsequently, construct optimization experiments revealed that expression of RpMCD, AvMCD, and CmMCD construct containing residues 8–451, 1–448, and 57–473, respectively, yielded soluble protein in each case without noticeable protein aggregation. The pET expression vectors for these constructs (NESG RpR127-8-451-21.13, NESG RiR35-1-448-21.13, and NESG ReR178-25-448-28) have been deposited in the Protein Structure Initiative Materials Repository (http://psimr.asu.edu).

Selenomethionyl RpMCD, AvMCD, and native CmMCD were purified by standard methods. Cell pellets were resuspended in lysis buffer (50 mM Tris [pH 7.5], 500 mM NaCl, 40 mM imidazole, and 1 mM Tris-(2-carboxyethyl)phosphine) and disrupted by sonication. The resulting lysate was clarified by centrifugation at 26,000 × *g* for 45 min at 4°C. The supernatant is then loaded onto an ÄKTAxpress system (GE Healthcare), and a two-step automated purification protocol is performed, comprised of a Ni-affinity column (HisTrap HP, 5 ml) and a gel filtration column (Superdex 75 26/60, GE Healthcare) in a linear series. A buffer containing 10 mM Tris (pH 7.5), 100 mM NaCl, 5 mM dithiothreitol (DTT), and 0.02% (w/v) NaN_3_ is used for gel filtration. The purified Se-Met labeled RpMCD, AvMCD, and native CmMCD were concentrated to 11, 8, and 10 mg/ml, respectively, flash frozen in aliquots, and used for crystallization screening. Sample purity (>95%) and molecular weight were verified by SDS-PAGE and MALDI-TOF mass spectrometry, respectively.

### Protein Crystallization

Purified HsMCD (SER quintuple mutant) was concentrated to 10 mg/ml in a buffer containing 5 mM 4-(2-hydroxyethyl)-1-piperazineethanesulfonic acid (HEPES) (pH 7.5), 100 mM NaCl, 1% (v/v) glycerol,l and 5 μM decanoyl-CoA. Crystals were obtained by sitting-drop vapor diffusion at room temperature by incubating protein in a 2:1 ratio with a precipitant containing 10% (w/v) polyethylene glycol (PEG) 20,000 and 0.1 M 2-(N-morpholino)ethanesulfonic acid (pH 6.0). The crystals belong to space group *C*222_1_, with a dimer of HsMCD in the asymmetric unit. The tetramer is generated through a crystallographic 2-fold axis.

The purified Se-Met-labeled RpMCD, AvMCD, and native CmMCD were crystallized using microbatch method at 18°C. In the case of RpMCD and AvMCD, 2 μl of the protein solution containing 10 mM Tris (pH 7.5), 100 mM NaCl, 5 mM DTT, and 0.02% NaN_3_ were mixed with 2 μl of the precipitant solution consisting of 0.1 M magnesium nitrate, 100 mM Tris (pH 8.5), and 33% (v/v) PEG 400 for RpMCD and 200 mM ammonium sulfate and 20% (w/v) PEG3350 for AvMCD. For CmMCD, 2 μl of the protein in a buffer consisting of 20 mM Tris (pH 7), 250 mM NaCl, 5% (v/v) glycerol, and 3 mM malonyl-CoA were mixed with a crystallization cocktail containing 160 mM magnesium chloride, 80 mM Tris (pH 8.5), 24% (w/v) PEG 4000, 20% (v/v) glycerol, and 3% (v/v) ethanol. The RpMCD and AvMCD crystals were cryoprotected by supplementing their respective crystallization cocktail with 20% (v/v) ethylene glycol and 20% (v/v) glycerol, respectively. No cryoprotecting solution was added into the crystallization cocktail containing CmMCD crystals for data collection at 100 K.

Crystals of RpMCD, AvMCD, and CmMCD belong to space group *P*2_1_2_1_2, *I*4_1_22 and *C*2, respectively, with four, one, and two molecules in the crystallographic asymmetric unit.

### Structure Determination and Refinement

For HsMCD, the structure was solved by multiple isomorphous replacement with anomalous scattering phasing. HsMCD crystals were derivatized with thimerosal or K_2_PtCl_4_ by 20 min incubation in reservoir solution supplemented with 5 mM of the respective heavy atom compound. X-ray diffraction data were collected at the Diamond Light Source beamlines IO2 and IO3 and processed and scaled with XDS (Kabsch, 2010) and Scala (Collaborative Computational Project, 1994), respectively. SHELXD (Sheldrick, 2008) identified three heavy atom sites in the mercury derivative. After including both derivatives in SHARP (Vonrhein et al., 2007) and subsequent density modification with SOLOMON (Abrahams and Leslie, 1996), substantial parts of the model were automatically built with BUCANEER (Cowtan, 2006). Manual model rebuilding was carried out with Coot (Emsley and Cowtan, 2004) and structure refinement with BUSTER (Global Phasing). No ligand electron density was observed in the active site. Residues 60–65, 115 and 116, 276–281, and 344–371, which represent surface-exposed regions in the structure, are disordered and not modeled.

The structure of RpMCD was determined by a single-wavelength anomalous diffraction data set to resolution 3.1 Å, which was collected at the peak absorption wavelength of selenium at the X6A beamline of the National Synchrotron Light Source. The diffraction images were processed with the HKL package (Otwinowski and Minor, 1997), and the selenium sites were located with the program SHELX (Sheldrick, 2008). SOLVE/RESOLVE was used for phasing the reflections and automated model building (Terwilliger, 2003). The majority of the model was built manually with the program XtalView (McRee, 1999). The structure refinement was performed with CNS 1.3 (Brünger et al., 1998).

The model thus obtained for RpMCD was used as a search model for structure determination of another data set of RpMCD to resolution 2.7 Å. The model was subsequently used to determine structures of CmMCD and AvMCD to resolution 2.3 Å and 3.1 Å, respectively, using the molecular replacement method implemented in the program Molrep (Vagin and Teplyakov, 2000). The data processing and refinement statistics are summarized in Table 1.

### Decarboxylase Activity Measurement

MCD catalytic activity was determined following a published protocol (Kolattukudy et al., 1981). For HsMCD, the following reagents were added to a total of 100 μl in a 96 well plate: 50 mM HEPES (pH 7.5), 1 mM dithiothreitol, 5 mM L-malate, 1 mM nicotinamide adenine dinucleotide (NAD)^+^, 0.1 mM reduced NAD, 1.925 U malate dehydrogenase (Sigma-Aldrich), 0.4 U citrate synthase (Sigma-Aldrich), 100-1000 nM HsMCD protein, and various concentrations (0 μM–500 μM) of malonyl-CoA. Absorbance changes at 340 nm were measured for 30 min and linear velocity used to calculate enzyme activity using GraphPad Prism (v.5.01).

### Analytical Ultracentrifugation

Sedimentation velocity (SV) experiments were performed in a Beckman Optima XL-I analytical ultracentrifuge (Beckman Instruments) using AnTi-50 rotor. Experiments were conducted at 30,000 rpm and 4°C using absorbance detection and cells loaded with 50 μM HsMCD in 10 mM HEPES (pH 7.5) and 150 mM NaCl. SV data were analyzed using SEDFIT (Schuck, 2000), while sedimentation coefficients, *s*, were calculated with SEDNTERP (Laue et al., 1992) version 1.09.

### Analytical Gel Filtration

Analytical gel filtration was performed on a Superdex 200 HiLoad 10/30 column (GE Healthcare) pre-equilibrated with 10 mM HEPES (pH 7.5) and 150 mM NaCl at a flow rate of 0.3 ml/min.

### Electron Microscopy

We studied the HsMCD assembly by negative staining electron microscopy and single particle analysis. Data were collected on a FEI F20 field emission gun microscope, equipped with an 8k × 8k charge-coupled device camera. Images were collected under low dose mode at a magnification of 50,000X at a final sampling of 1.6 Å/pixel at the specimen level. Single particle images were selected interactively using the Boxer program from the EMAN package (Ludtke et al., 1999). Image processing was performed using the IMAGIC-5 package (van Heel et al., 1996), and the single particle images were analyzed by multivariate statistical analysis. Selected class averages were used to calculate a starting 3D volume by common lines using the Euler program in the IMAGIC-5 package with no symmetry imposed. Manual fitting of the HsMCD tetramer was performed with UCSF Chimera (Goddard et al., 2007).

## Figures and Tables

**Table 1:** Summary of Crystallographic Information

Structure	HsMCD	RpMCD	AvMCD	CmMCD
Space group	*C*222_1_	*P*2_1_2_1_2	*I*4_1_22	*C*2
Unit cell parameters (*a*, *b*, *c*, α, β, γ)	95.6, 175.3, 151.8, 90, 90, 90	141.5, 159.8, 108.6, 90, 90, 90	100.4, 100.4, 242.7, 90, 90, 90	191.0, 69.4, 74.4, 90, 103.8, 90
Resolution range for refinement (Å)^a^	30–2.8 (2.9–2.8)	30–2.7 (2.8–2.7)	30–3.1 (3.2–3.1)	30–2.3 (2.4–2.3)
Number of observations	495,940	627,249	110,903	163,015
*R*_merge_ (%)	12.5 (106.4)	6.0 (61.2)	10.5 (55.1)	6.3 (44.1)
Redundancy	5.0 (5.0)	4.7 (4.4)	5.2 (4.8)	3.7 (3.5)
I/σI	8.4 (1.6)	25.2 (2.4)	15.9 (2.6)	23.0 (2.7)
Number of reflections	31,694	123,627	19,052	37,613
Completeness (%)	100 (100)	95 (85)	89 (70)	89 (72)
*R* factor (%)	21.2 (25.6)	22.5 (34.0)	22.0 (26.1)	23.9 (28.6)
Free *R* factor (%)	25.5 (29.5)	27.9 (38.3)	29.1 (34.1)	28.6 (33.3)
rms deviation in bond lengths (Å)	0.010	0.007	0.009	0.007
rms deviation in bond angles (°)	1.1	1.3	1.4	1.2

a The numbers in parentheses are for the highest resolution shell.

**Table 2:** Summary of Kinetic Parameters on Human MCD

Enzyme	*K*_m_ (μM)	*k*_cat_ (s^−1^)	*k*_cat_/*K*_m_ (M^−1^s^−1^)
Wild-type HsMCD	38 ± 12	33 ± 2 (1)^a^	8.7 × 10^5^ (1)
Quintuple SER mutant	58 ± 17	45 ± 4 (0.73)	7.8 × 10^5^ (1.1)
H423N	32 ± 4	4.7 ± 0.1 (7.0)	1.4 × 10^5^ (6.2)
S329A	19 ± 4	0.30 ± 0.01 (110)	1.5 × 10^4^ (58)
Y456S	132 ± 19	44 ± 2 (0.75)	3.3 × 10^5^ (2.6)
S290F	37 ± 5	15 ± 1 (2.2)	4.1 × 10^5^ (2.1)

a The ratio for values between the wild-type and mutant enzymes are given in the parentheses.

## Accession Numbers

The PDB accession numbers for HsMCD, RpMCD, AvMCD, and CmMCD reported in this paper are 2YGW, 4KSA, 4KSF, and 4KS9, respectively.

## Supplemental Information

Document S1. Supplemental Results and Discussion, Figures S1–S3, and Table S1
