# Supplementary material for: Crystal Structures of Malonyl-Coenzyme A Decarboxylase Provide Insights into Its Catalytic Mechanism and Disease-Causing Mutations
Source: Structure. 2013 Jul 2;21(7):1182–92. doi: 10.1016/j.str.2013.05.001 (PMC3701320; doi:10.1016/j.str.2013.05.001)
Supplement: Document S1. Supplemental Results and Discussion, Figures S1–S3, and Table S1 [file mmc1.pdf]

Structure, Volume *21*

Supplemental Information

Crystal Structures of Malonyl-Coenzyme A Decarboxylase Provide Insights into Its Catalytic Mechanism and Disease-Causing Mutations

D. Sean Froese, Farhad Forouhar, Timothy H. Tran, Melanie Vollmar, Yi Seul Kim, Scott Lew, Helen Neely, Jayaraman Seetharaman, Yang Shen, Rong Xiao, Thomas B. Acton, John K. Everett, Giuseppe Cannone, Sriharsha Puranik, Pavel Savitsky, Tobias Krojer, Ewa S. Pilka, Wasim Kiyani, Wen Hwa Lee, Brian D. Marsden, Frank von Delft, Charles K. Allerston, Laura Spagnolo, Opher Gileadi, Gaetano T. Montelione, Udo Oppermann, Wyatt W. Yue, and Liang Tong

Inventory of Supplemental Information

Fig. S1, related to Fig. 2
Fig. S2, related to Fig. 3
Fig. S3, related to Fig. 5
Table S1, related to Fig. 6
Text
References

A

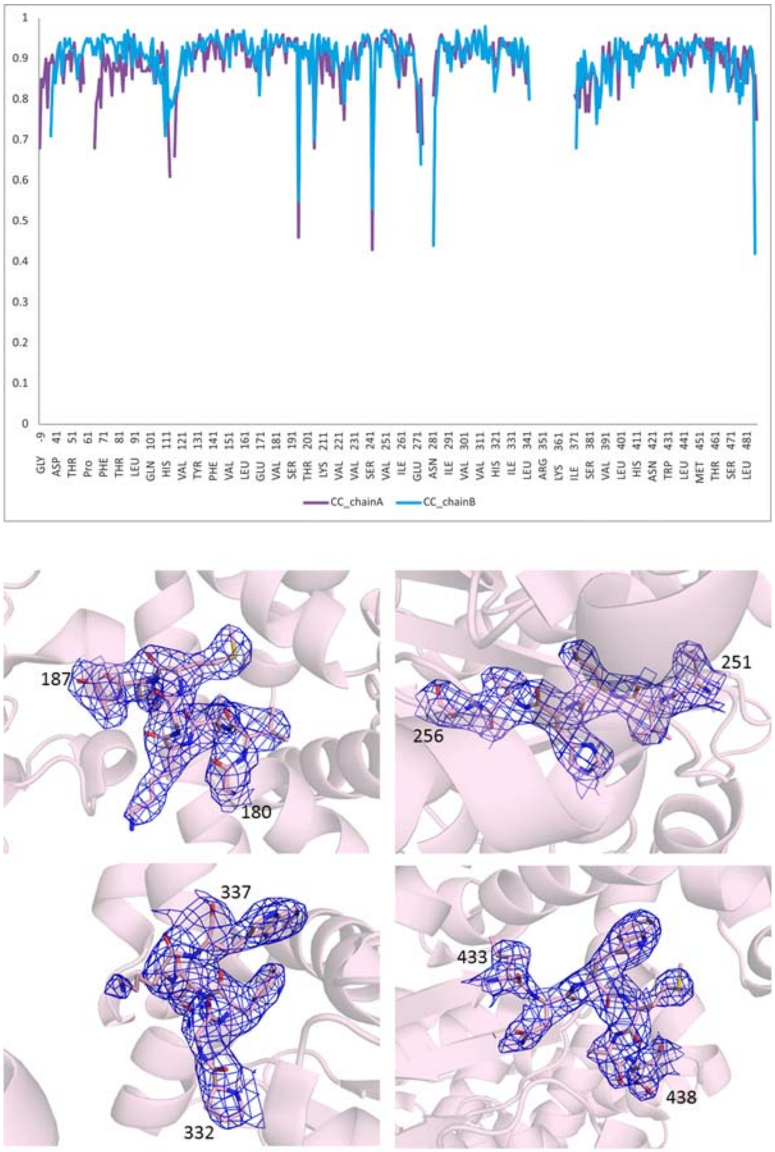
1
0.9
0.8
0.7
0.6
0.5
0.4
0.3
0.2
0.1
0
GLY -9
ASP 41
THR 51
Pro 61
PHE 71
THR 81
LEU 91
GLN 101
HIS 111
VAL 121
TYR 131
PHE 141
VAL 151
LEU 161
GLU 171
VAL 181
SER 191
THR 201
LYS 211
VAL 221
VAL 231
SER 241
VAL 251
ILE 261
GLU 271
ASN 281
ILE 291
VAL 301
VAL 311
HIS 321
ILE 331
LEU 341
ARG 351
LYS 361
ILE 371
SER 381
VAL 391
LEU 401
HIS 411
ASN 421
TRP 431
LEU 441
MET 451
THR 461
SER 471
LEU 481
CC_chainA
CC_chainB
187
180
256
251
337
332
433
438

B

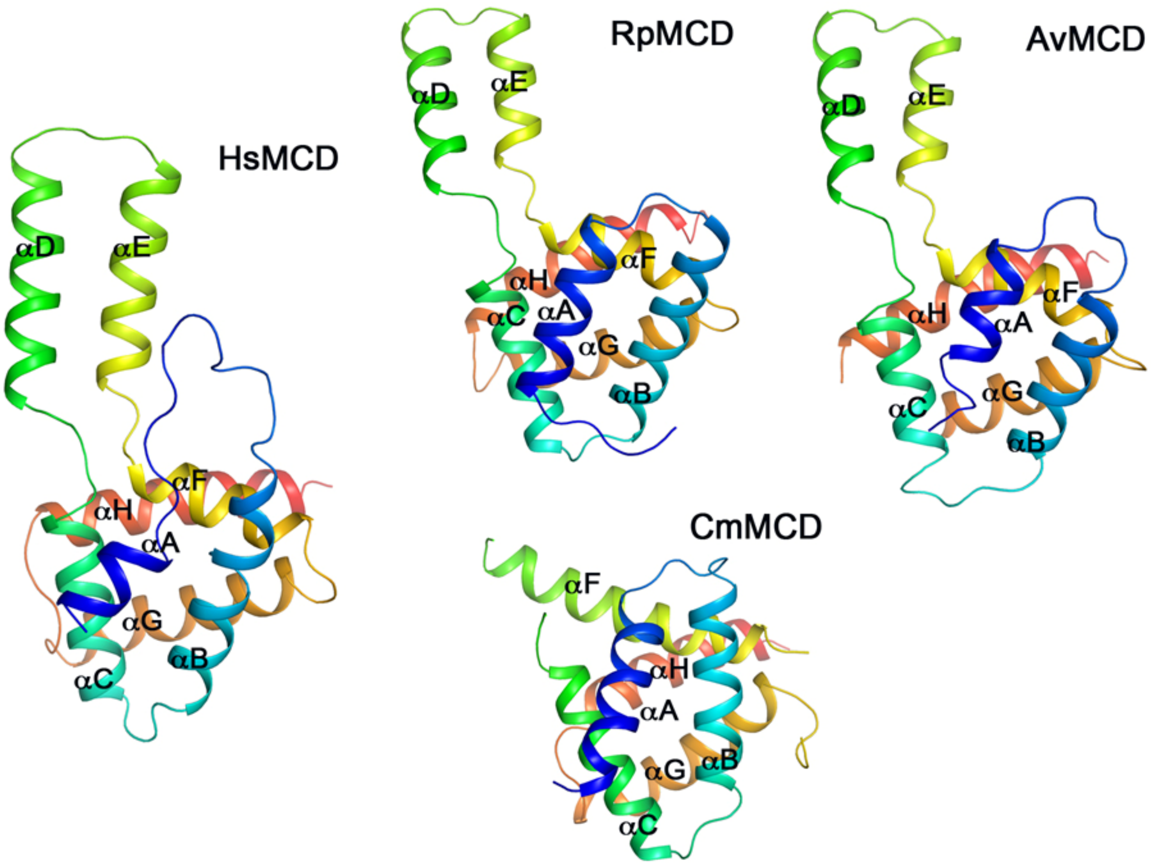
RpMCD
αD
αE
αF
αH
αC
αA
αG
αB
AvMCD
αD
αE
αF
αH
αA
αC
αG
αB
HsMCD
αD
αE
αF
αH
αA
αG
αC
αB
CmMCD
αF
αH
αA
αG
αB
αC

C

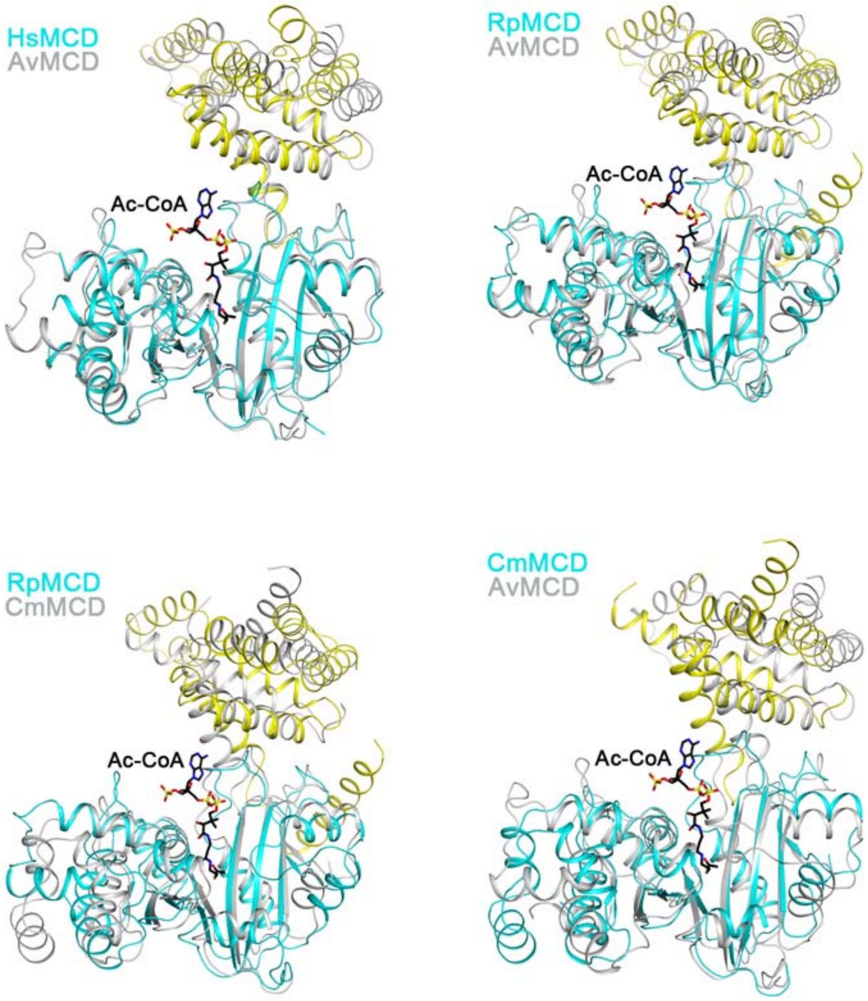

Fig. S1, related to Fig. 2.

A. (***Top***) Plot of real-space correlation coefficient (CC) for the two chains of HsMCD structure. The majority of the residues has CC greater than 0.9. Regions of poorer fit correspond to disordered/missing surface loops or alternate conformations. (***Bottom***) Quality of the HsMCD electron density map. $2F_o$-F_c map contoured at 1.2 σ is shown in blue mesh for four representative regions of the structure.

B. Schematic drawings of the structures of the N-terminal helical domain of MCD. The structures are colored from blue at the N-terminus to red at the C-terminus.

C. Overlay of MCD monomer structures. The overlay is based on the catalytic domain. One monomer is shown in color, and the other in gray. The catalytic domain of AvMCD is most similar to that of HsMCD.

A

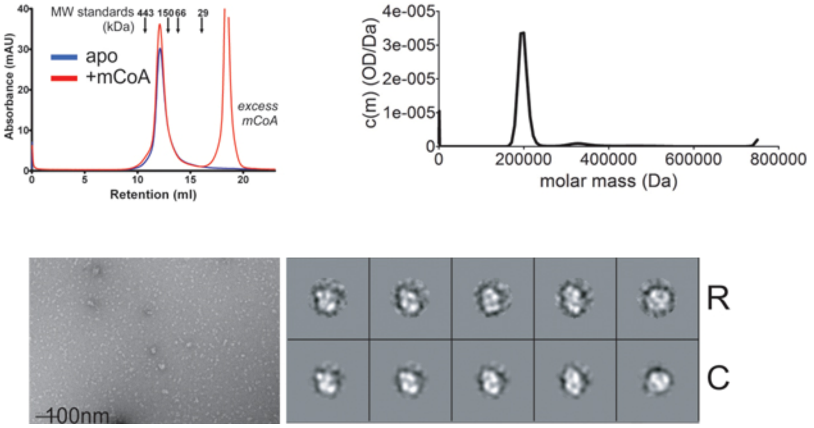
MW standards (kDa)
443
150
66
29
apo
+mCoA
excess mCoA
Absorbance (mAU)
Retention (ml)
c(m) (OD/Da)
4e-005
3e-005
2e-005
1e-005
0
0
200000
400000
600000
800000
molar mass (Da)
100nm
R
C

B

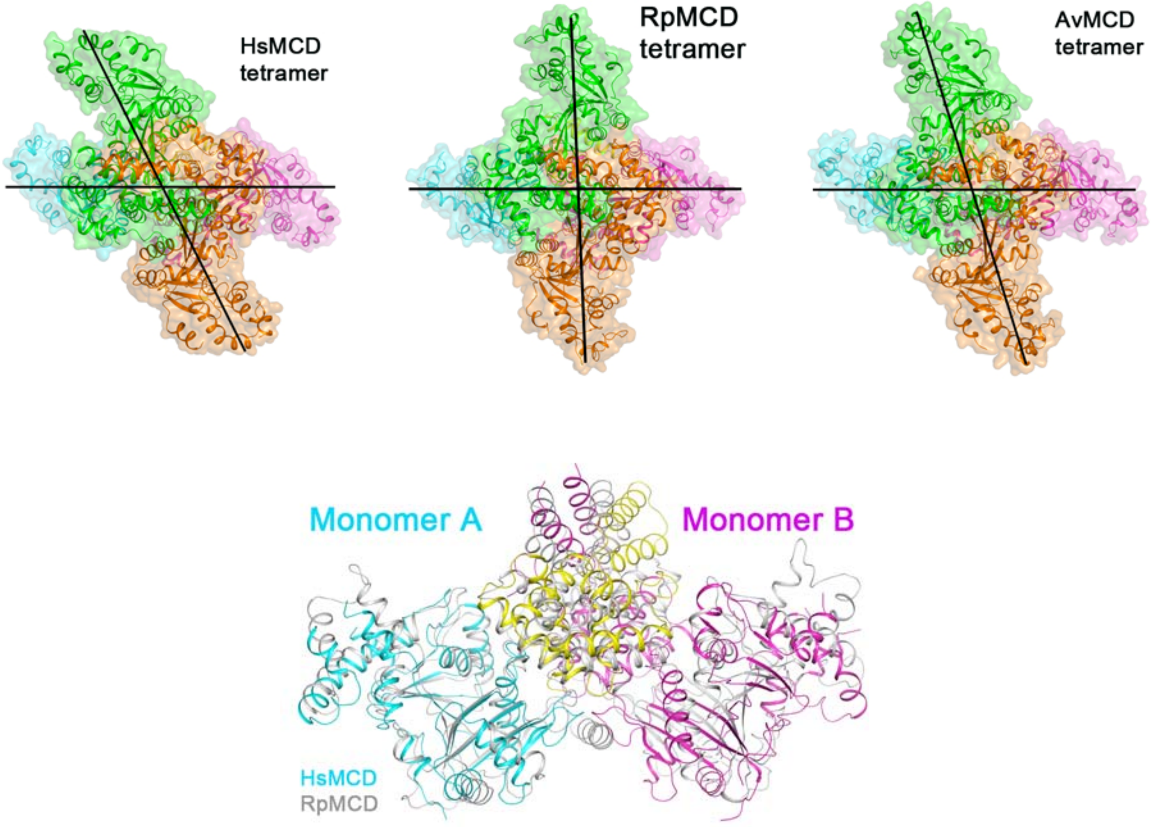
HsMCD tetramer
RpMCD tetramer
AvMCD tetramer
Monomer A
Monomer B
HsMCD
RpMCD

C

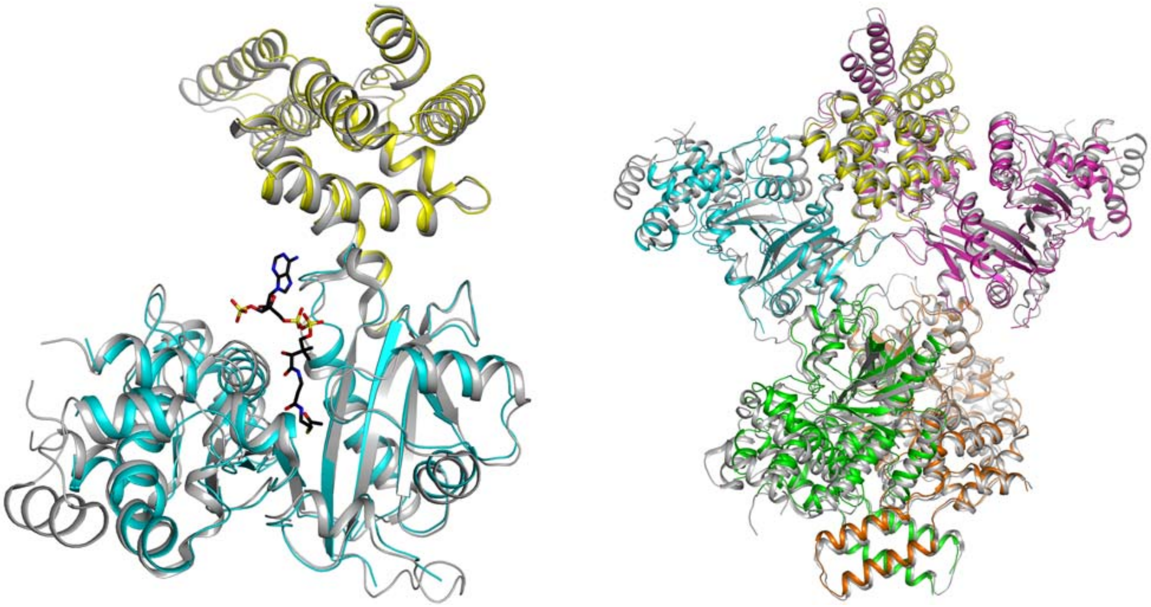

Fig. S2, related to Fig. 3.

A (***Top left***) Gel filtration chromatography of HsMCD in the apo form (blue) and supplemented with malonyl-CoA (mCoA, red) reveals a tetramer. Arrows indicate retention volumes of molecular weight standards. (***Top right***) Analytical ultracentrifugation shows HsMCD exhibits a narrow sedimentation coefficient distribution estimated to be a tetramer of ~200 kD. (***Bottom***) Left Panel: Representative electron microscopy image of HsMCD. The scale bar shown is 100 nm. Right Panel: Representative examples of 2D classifications (C) and re-projections (R) from the electron micrograph. Box size is 410 Å.

B (***Top***) Differences in the relative orientations of the dimers in the tetramers of HsMCD, RpMCD and AvMCD. The plane of the dimer in the back (cyan and magenta) is along the horizontal direction (indicated by the horizontal black line). The plane of the second dimer (green and orange, in the front) makes different angles in the three tetramers. (***Bottom***) Overlay of the structures of HsMCD (in color) and RpMCD (in gray) dimers. The overlay is based on the catalytic domain of monomer A.

C Overlay of the structures of HsMCD monomer (***left***) and tetramer (***right***), reported here (in color) and by (Aparicio et al., 2013) (in gray).

A

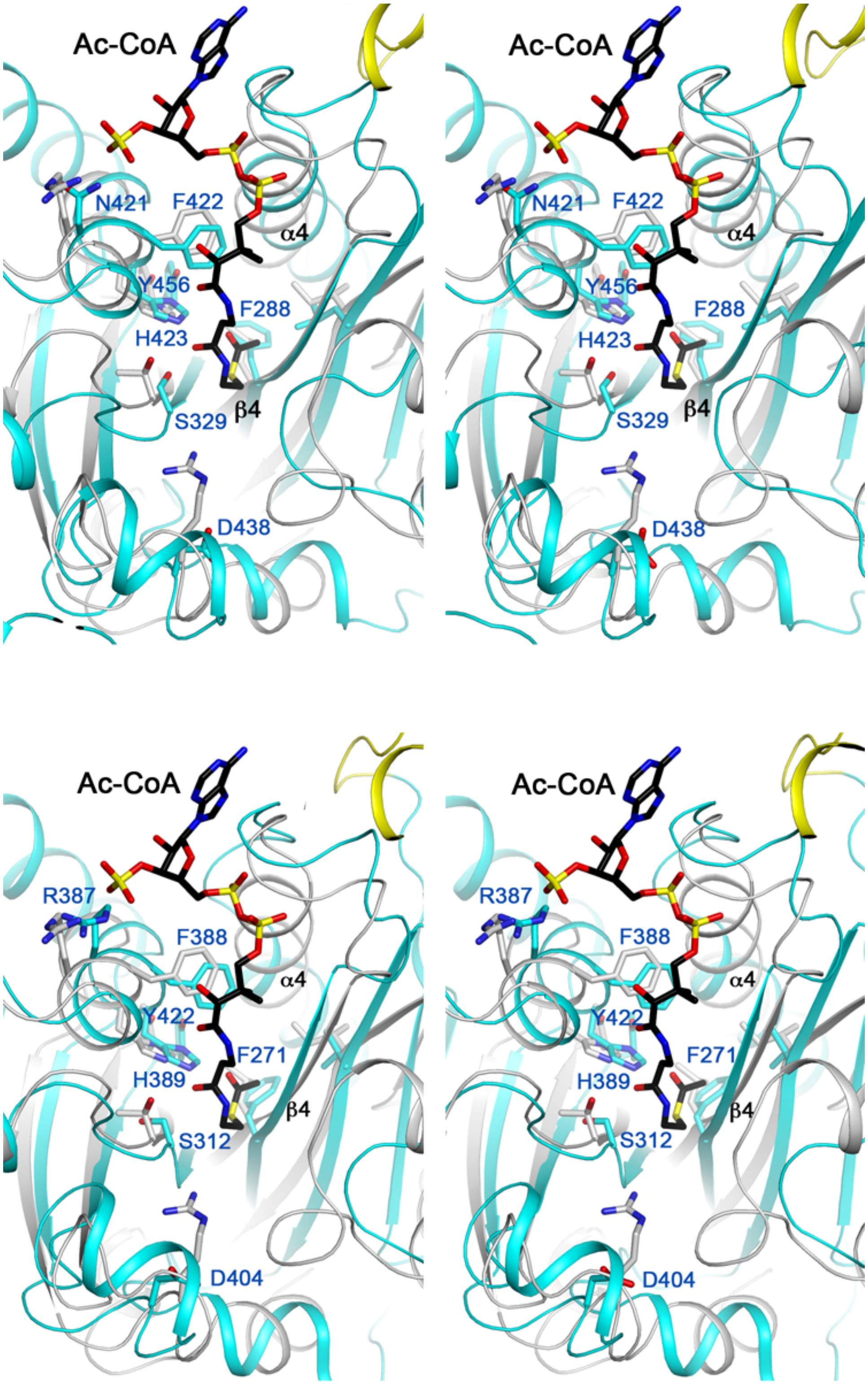
Ac-CoA
N421
F422
α4
Y456
F288
H423
S329
β4
D438
Ac-CoA
N421
F422
α4
Y456
F288
H423
S329
β4
D438
Ac-CoA
R387
F388
α4
Y422
F271
H389
β4
S312
D404
Ac-CoA
R387
F388
α4
Y422
F271
H389
β4
S312
D404

B

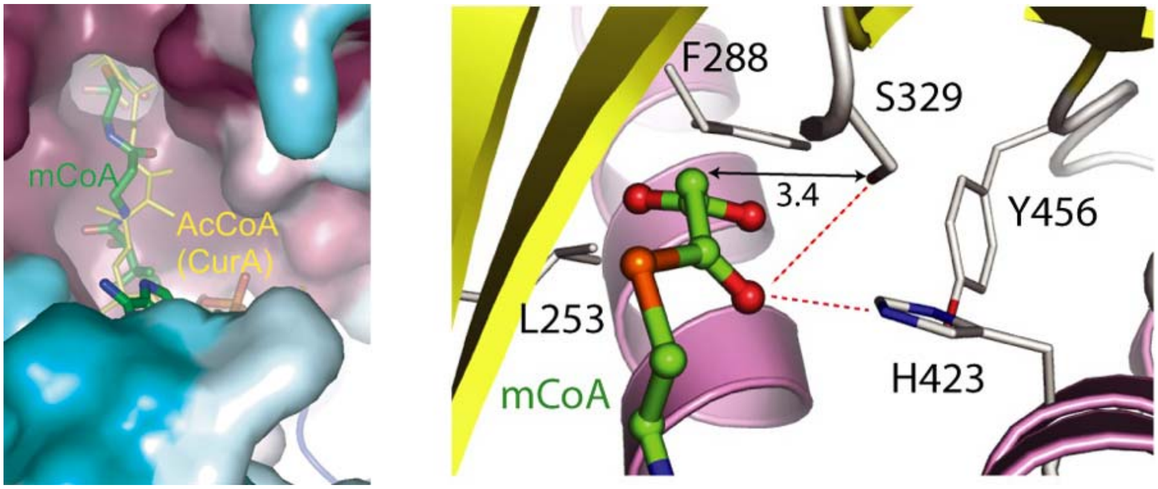

Fig. S3, related to Fig. 5.

A (***Top***) Overlay of the active site region of HsMCD (in cyan) and CurA (gray). Residues in HsMCD are labeled. (***Bottom***) Overlay of the active site region of RpMCD (in cyan) and CurA (gray). Both panels are in stereo view.

B Structural model of HsMCD docked *in silico* with malonyl-CoA (mCoA). (***Left***) Surface representation of the substrate channel showing in sticks the docked mCoA as well as acetyl CoA (AcCoA) from the CurA structure. Surface is coloured on the basis of sequence conservation among MCD homologues (magenta, most conserved; cyan, least conserved). (***Right***) Active site showing residues (in sticks) that can interact with the docked mCoA (hydrogen bonds, red dashed lines; distance in angstrom, black line).

Table S1, related to Fig. 6.
List of missense pathogenic mutations of HsMCD

No.	Mutation	Conservation	Location/proposed consequence
1	G3D	variable	Affect protein targeting?
2	M40T	conserved	N-terminal methionine for the mitochondrial isoform; Affect protein targeting?
3	A69V	variable	N-domain helix αB
4	L161P	conserved	N-domain helix αG
5	S290F	conserved	GNAT core, strand β2; in active site
6	G300V	conserved	GNAT core, linker between β4 and following helix; close to entrance of substrate channel
7	L307R	conserved	GNAT core, helix following β4; close to active site
8	W384C	variable	GNAT core, in a 3_{10} helix
9	S440I	conserved	GNAT core
10	Y456S	invariant	GNAT core, strand β7; hydrogen bond to catalytic residue His423
11	S477F	conserved	GNAT core

Supplementary text

An N-terminal helix in the central cavity of RpMCD tetramer

The expression construct for RpMCD covered residues 8-451 of this enzyme. Residues 10-29 form a helix (αA', Fig. 2B) and are located in the center of the tetramer (Fig. 3C). However, only two of the four helices of the tetramer are observed, as there is not sufficient space in the center of the tetramer to accommodate all four helices. In fact, the helix is situated on a two-fold axis of the tetramer (Fig. 3C). In an attempt to assess the importance of these residues for the tetramerization of RpMCD, we deleted the first 39 residues, but the resulting protein was not expressed in *E. coli*.

At the C-terminus, the expression construct of RpMCD lacked the last 25 residues of the protein. They are hydrophilic and poorly conserved among the MCDs (Fig. 1), suggesting that they are likely to be flexible. This C-terminal segment for HsMCD is much shorter, and it also contains the peroxisome targeting signal (SKL).

In silico substrate docking of HsMCD

The refined HsMCD structure was used as receptor for *in silico* docking with the substrate malonyl-CoA. Docking was performed with fully flexible ligands and potential grid maps representing five different features of the receptor, as implemented in the ICM software (Totrov et al., 1997). No restraints were introduced to receptor or ligand to allow full exploration of the active site, with the exception of thioester carbonyl oxygen atom. This atom was tethered to the oxygen atom of the β-alanine carbonyl of a bound acetyl-CoA (product of malonyl-CoA decarboxylation) in the CurA co-crystal structure (Gu et al., 2007), which has been superimposed to the crystal structure of HsMCD. The best poses were visually screened and selected if distance requirements for catalysis were found to be acceptable for atoms involved in the proposed catalytic mechanism. The docking result is shown in Fig. S6. The docked malonyl-CoA binds in a similar manner to that of acetyl-CoA in the CurA structure.

References

Aparicio, D., Perez-Luque, R., Carpena, X., Diaz, M., Ferrer, J.C., Loewen, P.C., and Fita, I. (2013). Structural asymmetry and disulfide bridges among subunits modulate the activity of human malonyl-CoA decarboxylase. J Biol Chem *288*, 11907-11919.

Gu, L. et al. GNAT-like strategy for polyketide chain initiation. *Science 318*, 970-4 (2007).

Totrov, M. & Abagyan, R. Flexible protein-ligand docking by global energy optimization in internal coordinates. *Proteins* Suppl 1, 215-20 (1997).
